# Enzymes Present in Neutrophil Extracellular Traps May Stimulate the Fibrogenic PGF_2α_ Pathway in the Mare Endometrium

**DOI:** 10.3390/ani11092615

**Published:** 2021-09-06

**Authors:** Maria Rosa Rebordão, Ana Amaral, Carina Fernandes, Elisabete Silva, Karolina Lukasik, Anna Szóstek-Mioduchowska, Pedro Pinto-Bravo, António Galvão, Dariusz J. Skarzynski, Graça Ferreira-Dias

**Affiliations:** 1 CIISA—Centro de Investigação Interdisciplinar em Sanidade Animal, Faculdade de Medicina Veterinária, Universidade de Lisboa, 1300-477 Lisboa, Portugal; milorebordao@gmail.com (M.R.R.); nita.amaral@gmail.com (A.A.); fachica@hotmail.com (C.F.); elisabetesilva@fmv.ulisboa.pt (E.S.); 2 Polytechnic Institute of Coimbra, College of Agriculture, 3045-601 Coimbra, Portugal; pbravo@esac.pt; 3 Department of Reproductive Immunology and Pathology, Institute of Animal Reproduction and Food Research of PAS, 10-748 Olsztyn, Poland; k.lukasik@pan.olsztyn.pl (K.L.); a.szostek-mioduchowska@pan.olsztyn.pl (A.S.-M.); a.galvao@pan.olsztyn.pl (A.G.); d.skarzynski@pan.olsztyn.pl (D.J.S.)

**Keywords:** neutrophil extracellular traps, fibrosis, endometrium, endometrosis, horse, PGF_2α_, PGF_2α_ receptor

## Abstract

**Simple Summary:**

Endometrosis is a fibrotic disease in mare endometrium whose pathological mechanisms remain obscure. Prostaglandin (PG)F_2α,_ despite modulating reproductive physiological processes, may also provoke local pathological collagen deposition (fibrogenesis). Neutrophil extracellular traps (NETs) released during inflammation have been linked to fibrogenesis in several tissues. We have previously shown that enzymes found in NETs increase in vitro collagen production in mare endometrium. In this study, activation of PGF_2α_-pathway in equine endometrial explants challenged in vitro by enzymes found in NETs is shown. Our results indicate that both endocrine microenvironment (estrous cycle phase) and healthy or pathological conditions of endometrial tissues play an important role in PGF_2α_-pathway activation. In the endometrium of the follicular phase, we have observed both high production of PGF_2α_ and/or PGF2α receptor gene transcription under the action of enzymes found in NETs, both conditions associated with fibrogenesis in other tissues. Nevertheless, transcription of the PGF_2α_ receptor gene does not appear to be hormone-dependent, albeit their levels seem to be dependent on endometrial category in the mid-luteal phase. This study suggests that enzymes existing in NETs may instigate changes on PGF_2α_ mediators, which may become an additional mechanism of fibrogenesis in mare endometrium.

**Abstract:**

Endometrosis, a fibrotic disease of mare endometrium, impairs uterine function. Prostaglandins (PG), despite modulating reproductive physiological functions, may also cause local pathological collagen deposition (fibrogenesis). We have previously shown that neutrophil extracellular traps (NETs) may also favor mare endometrosis. The aim of this study was to investigate the effect of enzymes present in NETs on PGF_2α_-pathway activation. Kenney and Doig’s type I/IIA and IIB/III mare endometria, from follicular phase (FLP) and mid-luteal (MLP) phase, were cultured in vitro in the presence of NETs enzymes (elastase, cathepsin-G or myeloperoxidase). Production of PGF_2α_ (EIA) and transcription (qPCR) of its synthases (*PTGS2*, *AKR1C3*) and receptor (*PTGFR*) genes were evaluated. PGF_2α_ and *PTGFR* were influenced by endometrial category and estrous cycle phase. In FLP endometrium, NETs enzymes induced both high PGF_2α_ production and/or *PTGFR* transcription. In MLP type I/IIA tissues, down-regulation of *PTGFR* transcripts occurred. However, in MLP type IIB/III endometrium, high levels of *PTGFR* transcripts were induced by NETs enzymes. As PGF2α-pathway activation facilitates fibrogenesis in other tissues, PGF2α may be involved in endometrosis pathogenesis. In the mare, the endocrine microenvironment of healthy and pathological endometrium might modulate the PGF_2α_ pathway, as well as fibrosis outcome on endometrium challenged by NETs enzymes.

## 1. Introduction

Neutrophil extracellular traps (NETs) are big DNA-associated molecule complexes of nucleic and cytoplasmic proteins, each one of them with strong antimicrobial and/or immunomodulating/proinflammatory properties, responsible for killing and/or trapping bacteria [1,2,3,4]. At the end of this active process called NETosis, neutrophil nuclear membrane ruptures and its granules decompose, allowing the chromatin to directly contact with most neutrophil proteins [5].

As a paradox, despite neutrophils being the first line of defense against the invading pathogenic bacteria, they may also be responsible for deleterious effects leading to several pathological processes [6]. The release of enzymes, such as elastase (ELA), cathepsin-G (CAT), or myeloperoxidase (MPO) may be one of the mechanisms by which neutrophils may contribute for a magnified inflammatory response and to tissue-specific injury with subsequent fibrosis formation [6,7,8,9,10]. In fact, it has been shown that NETs induce activation of lung fibroblasts and their differentiation into myofibroblasts, as well as collagen production [11].

The pathogenesis of tissue fibrosis is extremely complex and regulated in the inflamed tissues by intricate interactions of profibrotic cytokines, extracellular matrix (ECM) components, inflammatory cells, hormones, and fibroblasts [12]. In addition to cytokines, prostaglandins (PG) have been considered in fibrogenesis [13]. Prostaglandins are lipid autacoids derived from arachidonic acid (AA) that sustain homeostatic functions and mediate several pathogenic mechanisms, including the inflammatory response [14]. These mediators act locally, through specific receptors, due to their extremely short half-life in blood [13]. The release of AA from membrane glycerophospholipids is catalyzed by phospholipase A2 (PLA2) enzymes, and AA is sequentially metabolized to prostaglandin G2 (PGG2) and then to PGH2 by prostaglandin-endoperoxide synthase 1 (PTGS1) and/or PTGS2. While PTGS1 is constitutively expressed in most tissues and responsible for housekeeping functions, PTGS2 is regulated by several factors, such as cytokines, and supports a sustained production of PG [15]. Prostaglandin H2 is then converted to various bioactive PGs (thromboxane A2, PGD_2_, PGE_2_, PGF_2α_, and PG_I2_) [16]. The PG produced by a given cell largely depends on the expression profile of each prostaglandin synthase enzymes [17]. Prostaglandin F2α is produced through the reduction in PGH2, by aldoketoreductase 1C synthases (AKR1C3) [15]. Prostaglandins are ubiquitously produced and act locally in an autocrine or juxtacrine manner to elicit a diverse set of effects that modulate many physiological systems, as the reproductive one, but have also been implicated in a broad array of diseases [17]. Activation of a given PG receptor by its associated ligand might modulate varying responses in different cell types and tissues. Prostaglandin F_2α_ acting on its receptor (PTGFR) has been linked to fibrosis formation in other tissues, such as lung [13,18,19,20], heart [21,22], skin [23], and synovia [24]. 

Mare endometrial fibrosis (endometrosis) is the consequence of diverse insults that provoke degenerative changes of endometrial glands and surrounding stroma, characterized by periglandular arrangement of myofibroblasts and deposition of ECM components, mainly collagen [25,26]. Knowledge of the exact mechanisms behind endometrial fibrosis is lacking. As we have found the presence of NETs in the uterus of mares with infectious endometritis [27] and PGs are linked to inflammatory and fibrotic conditions [28], we hypothesized that NETS and the prostaglandin pathway may interplay in the development of mare endometrial fibrosis. Therefore, the aim of this study was to evaluate the PGF_2α_ pathway in mare endometrial explants challenged with enzymes found in NETs, by examining the expression and/or transcription of PGF_2α_, its synthases and its receptor genes. The influence of endometrial inflammatory/fibrotic lesions and of estrous cycle phase was also assessed. 

## 2. Materials and Methods

### 2.1. Collection of Mare’s Uteri and Blood and Experimental Design

Mare internal genitalia were collected *post-mortem* from randomly designated cyclic mares, of various breeds, aged from 3 to 25 years old, at the abattoir, during the reproductive season (May–September). The mares used in the present study were healthy and in good physical condition, as determined by *ante-mortem* veterinary inspection. Animal handling and slaughter conformed to European welfare (EFSA, AHAW/04–027) and Portuguese (DL 98/96, Art. 18) mandates. For confirming the reproductive status of the mares, venous blood samples were collected after slaughter, for progesterone (P4) determination in plasma.

Immediately after uteri collection, endometrial samples from the uterine horn ipsilateral to the ovary presenting either a growing follicle or a corpus luteum (CL) were carefully separated from the myometrium and washed in sterile phosphate-buffered saline (PBS) with antibiotics (100 mg/mL streptomycin—S9137; Sigma, St Louis, MO, USA and 100 IU/mL penicillin—P3032; Sigma-Aldrich). The macroscopic analysis of the ovaries was used to assign each animal to a specific phase of the estrous cycle. The follicular phase (FLP) was characterized by the presence of a follicle >35 mm in diameter and the absence of an active *corpus luteum* (CL), while in the mid luteal phase (MLP) a developed CL, with a solid trabeculated appearance [29] could also be associated with follicles 15–20 mm in diameter [30]. The macroscopic evaluation of the uteri was performed to confirm the absence of signs of endometritis, such as altered color of the mucosa, and/or increased mucus production. Endometrial smears were obtained with sterile swabs for further cytological analysis [31]. The healthy uteri collected were thus classified as belonging to the FLP (*n* = 10) or to the MLP (*n* = 10). Endometrial samples were then placed in either 4% buffered formaldehyde, for endometrial histological evaluation and classification; RNAlater (AM7020; Ambion, Applied Biosystems, Foster, CA, USA), for quantification of mRNA transcription; or kept intact in chilled (4 °C) Dulbecco’s modified Eagle’s medium (DMEM) and F-12 Ham medium (D/F medium; 1:1 (*v/v*); D-8900; Sigma) supplemented with antibiotics (100 mg/mL streptomycin, 100 IU/mL penicillin, and 2 mg/mL amphotericin—A2942; Sigma) for in vitro explant culture studies. Endometrial samples and blood were transported on ice to the laboratory within 1 h after sample collection. 

Endometria (*n* = 20) collected from different estrous cycle phases (*n* = 5 samples from each I/IIA or IIB/III endometrial group, from FLP and MLP), were further incubated with enzymes found in NETs. Qualitative and quantitative protein composition of NETs and the activities of those enzymes were the criteria used to select the concentrations of those proteins for in vitro explant incubations. ELA, CAT, and MPO were identified as the most abundant non-histone NETs proteins [32] and their activities were between 10 nM (0.3 μg/mL) to 100 nM (3.2 μg/mL) [33,34,35,36]. The MPO concentrations used in our in vitro studies are within the range of those detected in the uterine lumen of mares [37]. In addition, concentrations of each enzyme were determined by a preliminary dose response experiment (data not shown) performed in our laboratory. TGF-β1 production (as a putative fibrotic marker) by endometrial explants was evaluated in response to three concentrations of each protein: ELA (0.1, 0.5 or 1 μg/mL), CAT (0.1, 1 or 2.5 μg/mL), or MPO (0.1, 0.5, or 1 μg/mL). As the lowest concentration of ELA (0.1 μg/mL) did not stimulate a high secretion of TGF-β1, and the highest dose of CAT (2.5 μg/mL) and MPO (1 μg/mL) did not induce an additional increase in TGF-β1 production, further endometrial incubations with those proteins were performed with the two other tested concentrations.

### 2.2. Mare Endometrial Cytological and Histopathological Evaluation and Classification 

To evaluate the presence of neutrophils in the uteri, endometrial smears were stained with Diff-Quick (NC0851891; Fisher Scientific, Porto Salvo, Portugal). Endometria were considered as healthy, and used in tissue culture, when on average they presented less than two neutrophils per four light microscopic fields (Mag 400×) [31]. 

Histological sections of 5 μm formaldehyde fixed endometrial samples were stained with hematoxylin (05-06014E; Bio-Optica, Milan, Italy) and eosin (HT1103128; Sigma-Aldrich, St Louis, MO, USA). Each sample was examined for the presence of inflammatory cells, periglandular fibrosis, glandular distribution, and lymphatic lacunae, under light microscope (Leica DM500). Less than one neutrophil per field, at 400× magnification (in 5 random fields), was a further basis for considering that the uteri were free of inflammation [38]. Endometrial samples were classified according to Kenney and Doig´s [39], in category I, IIA, IIB, or III, corresponding to minimum, mild, moderate, or severe lesions of endometrial fibrosis, respectively. Endometria categorization was performed by two experienced researchers. All subsequent studies were completed after clustering the endometrial samples into two endometrial classification groups from FLP and MLP: (i) I/IIA group (*n* = 5), included type I (*n* = 3) and IIA (*n* = 2) endometria; (ii) IIB/III group (*n* = 5), comprised type IIB (*n* = 2) and type III (*n* = 3) tissues. The average (± SD) age of the mares in each group was as follows: in FLP I/IIA group (*n* = 5), 5.3 ± 3.5 years; in FLP IIB/III group (*n* = 5), 12.9 ± 4.5 years; in MLP I/IIA group (*n* = 5), 6.4 ± 3.6 years; and in MLP IIB/III group (*n* = 5), 13.8 ± 5.8 years.

### 2.3. Endometrial Explants In Vitro Culture

Tissue culture was performed as established in our previous studies [30,40] using strips of endometrium (*n* = 5 samples from each group - I/IIA and IIB/III) collected from each estrous cycle phase (FLP and MLP). After being carefully washed with PBS and antibiotics, approximately 20–30 mg of endometria was placed in cell culture medium of each well of a sterile 24 well cell culture plate (Eppendorf, #0030 722.116), for 1 h, at 37 °C in a humidified atmosphere (Biosafe Eco-Integra Biosciences, Chur, Switzerland; 5% CO_2_, 95% air) with gentle shaking (Titertek, Huntsville, AL, USA; 150 r.p.m.). Culture medium consisted of DME/F-12 Ham Medium (D/F medium; 1:1 (*v/v*); D-8900; Sigma) supplemented with bovine serum albumin (BSA; 0.1% (*w/v*); 735078; Roche Diagnostics, Mannheim, Germany), streptomycin (100 mg/mL), penicillin (100 IU/mL), and amphotericin (2 mg/mL).

The medium was changed after 1 h pre-incubation, and endometrial explants were cultured for further 24 h with: (i) medium—control; or (ii) elastase (ELA; 0.5 or 1 µg/mL; A6959, AppliChem GmbH, Darmstadt, Germany); (iii) cathepsin G (CAT; 0.1 or 1 µg/mL; A6942, AppliChem GmbH); (iv) myeloperoxidase (MPO; 0.1 or 0.5 µg/mL, A6969, AppliChem GmbH); or (v) oxytocin (OXT; 10^−7^ M) as positive control of endometrial PG secretory function [41]. Explants were incubated under the same conditions as pre-incubation and each treatment was performed in quadruplicate. When incubation time was over, culture medium was immediately used for alamarBlue^®^ (AB; DAL1100, ThermoFisher Scientific, Waltham, MA, USA) explant metabolic viability assessment. The remaining conditioned medium was collected to eppendorf’s with 1% of PG stabilizer (0.3 M ethylenediaminetetraacetic acid (EDTA) and 1% aspirin (A2093; Sigma) and kept at −80 °C for further PGF_2α_ determinations [42]. Conditioned explants were maintained in cryotubes with RNAlater^®^ at −80 °C, until its further use in gene transcription assessment.

### 2.4. Assessment of Endometrial Explants Viability

Metabolic viability of the explants was evaluated by AB, as previously described [43]. Briefly, fresh (non-incubated), treated and non-treated control explants incubated for 24 h and culture medium were incubated under the same conditions described above for an additional 4 h with 10% AB. Fluorescence values were read at 530 nm excitation/590 nm emission wavelengths using a fluorometer microplate reader (Synergy H1 Hybrid Reader, BioTek; Gene 5 software). The percentage of viability was obtained by calculating the percentage (%) of AB reduction per mg of endometrium [43].

In addition, as PGF_2__α_ response of non-treated and OXT treated explants suggests that the endometrial explants contain functional endometrial cells [42], this was also used as the criterion of their functionality and consequent viability.

### 2.5. Hormone Assays

Plasma P4 concentrations were measured in duplicate by a validated solid-phase Radioimmunoassay (RIA), without extraction, using a commercial kit (Coat-A-Count; Diagnostic Product Corporation, Los Angeles, CA, USA), and a Wallac (wizard 1470) counter, as previously described [44]. All samples were run in a single assay. The limit of detection of the assay was 0.02 ng/mL and the intra-assay coefficient of variation for all samples was 3.4%. Circulating P4 concentrations were used to help determine estrous cycle phase of the animals. Mares with plasma P4 concentrations >6 ng/mL were considered in MLP, while P4 values <1 ng/mL were indicative of FLP [45].

Culture medium PGF_2α_ was measured by direct enzyme immunoassay (EIA), as previously described [42]. The PGF_2α_ standard curve ranged from 0.19 ng/mL to 50 ng/mL and the intra- and inter-assay coefficients of variation were 8.5% and 10.7%, respectively. Hormone concentrations in culture media were normalized for mg of endometrium.

### 2.6. Gene Transcription Analysis

The mRNA expression levels of genes involved in the PGF_2α_ pathway, as prostaglandin endoperoxide-synthase 2 (*PTGS2*), F2α synthase (*AKR1C3*-aldo-keto reductase C3), PGF_2α_ receptor (*PTGFR*) was assessed by quantitative real time-PCR (qPCR). Extraction of RNA from endometrial explants was performed with the Total RNA Extraction and Purification kit (28704; Qiagen, Hilden, Germany) according to the manufacturer instructions, including the DNA-digestion step (RNase-free DNase Set; 50979254; Qiagen [46]. Quantification and assessment of mRNA quality were performed with Nanodrop (ND200C; Fisher Scientific) and by the identification of 28 S and 18 S mRNA bands in a 1.5% agarose gel and red staining (41003; Biotium, Hayward, CA, USA). One μg of total RNA was reverse transcribed with oligo (dT) primer (27-7858-01, GE Healthcare, Buckinghamshire, UK) using SuperScript™ III Reverse Transcriptase (18080093; Invitrogen, GIBCO BRL, Carlsbad, CA, USA) in a 20 μL reaction volume, and cDNA was stored at −20 °C.

Internet-based program Primer-3 [47] and Primer Premier software (Premier Biosoft Interpairs, Palo Alto, CA, USA) were used to design specific primers (Table 1). To prevent genomic DNA amplification, primers were designed on two different exons flanking one intron. Ribosomal protein L32 (RPL32) was used as reference gene, chosen from four potential reference genes initially considered [48], as its transcription was not affected by treatments. Primer concentrations were optimized to the minimum concentration: lowest cycle threshold ratio. qPCR was performed in duplicate wells in a StepOnePlus™ Real-Time PCR System (Applied Biosystems, Warrington, UK), using the universal cycling conditions: 10 min of pre-incubation at 95 °C, followed by 40 two-temperature cycles (15 s at 95 °C and 1 min at 60 °C). To confirm primers specificity, the melting curve analysis step was included (15 s at 95 °C, 30 s at 60 °C, and 15 s at 95 °C). Additionally, the identity of the PCR products was initially confirmed by DNA sequencing.

The qPCR reactions were performed using 6.5 µL of SYBR Green PCR Master Mix (Applied Biosystems), 80 nM for all primers except for PTGFR, which was used at 160 nM, 1 µL of cDNA in a total reaction volume of 13 µL. To assess the specificity of each amplicon, PCR products were run on a 2.5% agarose gel (BIO-41025; Bioline, Luckenwalde, Germany). Real-time PCR miner algorithm [49] was used to quantify relative mRNA expression levels. For each sample, the average cyclic threshold (Ct) was related to the primer efficiency level (E) using the equation [1/(1þE)Ct] [49]. Target gene transcription was then normalized against that of the reference gene and relative expression values were calculated. Relative mRNA levels of control samples were compared with treated explants data.

### 2.7. Statistical Analysis

Statistical analysis was performed using STATISTICA, Version 8 (StatSoft, Inc., Tulsa, OK, USA). Data regarding PGF_2α_ secretion of non-treated and OXT-treated endometrial explants, to assess functional capacity of incubated tissues, were evaluated by Student’s *t*-test. Data of endometrial explants viability and age of the mares were analyzed by One-way analysis of variance (ANOVA) followed by Dunnett’s *post hoc* test. These results are presented as the mean ± SD. Results of the relative mRNA expression and PGF_2α_ output of control and treated explants were assessed through general linear model’s factorial ANOVA, with the main effects of the phase of the estrous cycle, endometrial categories, explant treatments and their 2- and 3-way interactions. Post hoc LSD (least significant difference) tests were performed to compare means. Data are presented as the mean ± S.E.M., unless otherwise specified. Statistical significance was defined as *p* value less than 0.05.

## 3. Results

### 3.1. Viability of Endometrial Explants

Endometrial explants showed high metabolic activity and no differences in the percentage of AB reduction were observed between fresh (non-incubated; 98.1 ± 1.2%) and 24 h incubated control (non-treated) tissues (94.94 ± 3.9%) (*p* > 0.05). Similarly, after incubation of mare endometrial explants with ELA, MPO, and CAT, the percentage of difference in AB reduction between treated and non-treated control samples was not different (ELA 0.5 µg/mL = 94.62 ± 7.1%; ELA 1 µg/mL = 95.05 ± 7.5%; MPO 0.1 µg/mL = 97.79 ± 14.7%; MPO 0.5 µg/mL = 97.26 ± 12.3%; CAT 0.1 µg/mL= 95.52 ± 7.1%; CAT 1 µg/mL = 98.7 ± 10.6%) (*p* > 0.05). Furthermore, PGF_2__α_ response of explants to OXT treatment suggests that the endometrial explants contained functional endometrial cells. Secretion of PGF_2__α_ by endometrial explants after incubation with OXT (9.88 ± 4.6 ng/mg) was increased when compared with incubated non-treated tissues (4.4 ± 2.7 ng/mg) (*p <* 0.05).

### 3.2. Effects of NETs Enzymes on the PGF_2α_ Synthesis

To evaluate the effect of ELA, MPO, and CAT enzymes on endometrial PGF_2α_ secretory function, the mRNA levels of the *PTGS2*, *AKR1C3*, and *PTGFR* genes, as well as PGF_2α_ production were assessed. Additionally, the effect of the estrous cycle phase, endometrial category, and treatments on PGF_2α_ activity is presented in Table 2. For *PTGS2* mRNA levels, there were significant interactions between estrous cycle phases and treatments, and between endometrial category groups and treatments (*p* < 0.001; Table 2). Significant interactions between all variables and treatments were also observed for A*KR1C3* (*p* < 0.001) and *PTGFR* transcription (*p* < 0.001) and for PGF_2α_ production (*p* < 0.001) (Table 2).

**Figure 1:**
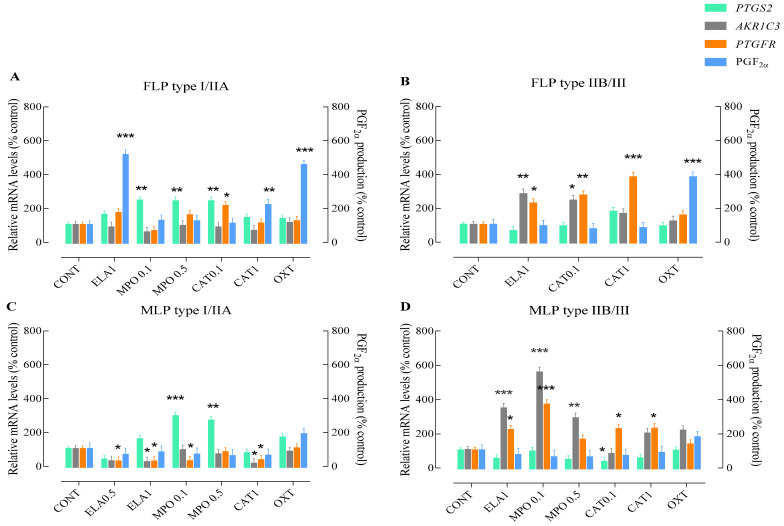
Relative *PTGS2*, *AKR1C3*, or *PTGFR* mRNA transcription and PGF_2α_ production in type I/IIA or type IIA/III follicular phase (FLP; **A**,**B**) and mid-luteal phase (MLP; **C**,**D**) mare endometrial explants after 24 h incubation with different doses of enzymes found in NETs. Elastase—ELA: 0.5 or 1 µg/mL; myeloperoxidase—MPO: 0.1 or 0.5 µg/mL; and cathepsin G—CAT: 0.1 or 1 µg/mL. All values are expressed as percentage of change from control (CONT—non-treated tissues). Oxytocin (OXT; 10^7^ M) was used as a PGF_2α_ positive control. Bars represent the least significant difference (LSD) means ± S.EM. Asterisks indicate significant differences from the respective control (* *p <* 0.05; ** *p* < 0.01; and *** *p <* 0.001).

In type I/IIA mare endometrial explants obtained from FLP incubated with the lowest concentration of ELA (0.5 µg/mL), no differences from the control group were detected for any of the studied PGF_2α_-pathway mediators (data not shown). Nevertheless, the highest tested concentrations of ELA (*p* < 0.001) or CAT (*p* < 0.05) increased PGF_2α_ production, compared to the non-treated tissues (Figure 1A). Treatment with both tested concentrations of MPO resulted in increased *PTGS2* transcripts (*p* < 0.01; Figure 1A), when compared to the control group. Endometrial incubation with the lowest concentration of CAT enhanced both *PTGS2* (*p* < 0.01) and *PTGFR* mRNA levels (*p* < 0.05; Figure 1A), compared to non-treated tissues. No differences were found in *AKR1C3* transcripts throughout the incubation period, regardless of NETs proteins (*p* > 0.05; Figure 1A). 

Incubation of FLP type IIB/III mare endometrial explants with the highest concentration of ELA enhanced *AKR1C3* (*p* < 0.01; Figure 1B) and *PTGFR* gene transcription (*p* < 0.05; Figure 1B), compared to the non-treated tissues. Similarly, treatment of endometrial explants with the lowest dose of CAT increased *AKR1C3* transcripts (*p* < 0.05; Figure 1B), and both concentrations of CAT up-regulated *PTGFR* gene transcription (CAT 0.1 µg/mL—*p* < 0.01; CAT 1 µg/mL—*p* < 0.001; Figure 1B). However, PGF_2α_ production levels were not affected by endometrial explant treatments, although they were up-regulated with OXT stimulation (*p* < 0.001; Figure 1B). No differences from the control group were observed in any of the studied mediators of the PGF_2__α_-pathway when tissues were challenged with the lowest concentration of ELA and both doses of MPO (data not shown).

In MLP type I/IIA mare endometrium, both concentrations of ELA treatment decreased *PTGFR* mRNA levels (*p* < 0.05; Figure 1C), while a decrease in *AKR1C3* transcripts was also noted with the highest dose of ELA (*p* < 0.05; Figure 1C), when compared to the control group. Although the lowest concentration of MPO also provoked a down-regulation of *AKR1C3* transcripts (*p* < 0.05; Figure 1C), the same type of endometrium responded to both concentrations of MPO by up-regulating *PTGS2* gene transcription (MPO 0.1 µg/mL—*p* < 0.001; MPO 0.5 µg/mL—*p* < 0.01; Figure 1C). In tissues treated with the lowest concentration of CAT, no differences from the control group were detected in any of the studied PGF_2α_-pathway mediators (data not shown). However, endometrial incubation with the highest tested concentration of CAT (*p* < 0.05) decreased both *AKR1C3* and *PTGFR* mRNA levels (*p* < 0.05; Figure 1C). Regardless of the treatment, no changes were observed in the production of endometrial PGF_2__α_ (*p* < 0.05; Figure 1C).

In MLP type IIB/III mare endometrial explants treated with the lowest concentration of ELA, no differences from the control group were detected in any of the studied parameters (data not shown). Tissue incubation with the highest concentration of ELA and the lowest dose of MPO up-regulated both *AKR1C3 (p* < 0.001) and *PTGFR (ELA* 1 µg/mL—*p* < 0.05; MPO 0.1 µg/mL—*p* < 0.001) mRNA levels (Figure 1D). However, in endometria treated with the highest dose of MPO, only an increase in *AKR1C3* transcripts was noted (*p* < 0.01; Figure 1D). The same type of endometrium responded to stimulation with the lowest concentration of CAT by down-regulating *PTGS2* (*p* < 0.05; Figure 1D), and up-regulating *PTGFR* (*p* < 0.05; Figure 1D) gene transcription. With the highest dose of CAT, an increase in *PTGFR* mRNA levels was also observed (*p* < 0.05; Figure 1D). No changes in PGF_2α_ production were observed after stimulation of endometrial explants with NETs enzymes (*p* > 0.05; Figure 1D).

### 3.3. Influence of the Endometrial Category on the PGF_2α_ Pathway Activation by NETs Enzymes

The influence of endometrial types (I/IIA vs. IIB/III) on the putative fibrogenic PGF_2α_ pathway triggered by enzymes found in NETs was evaluated. This analysis was performed comparing the same treatments within the same estrous cycle phase (FLP or MLP).

**Figure 2:**
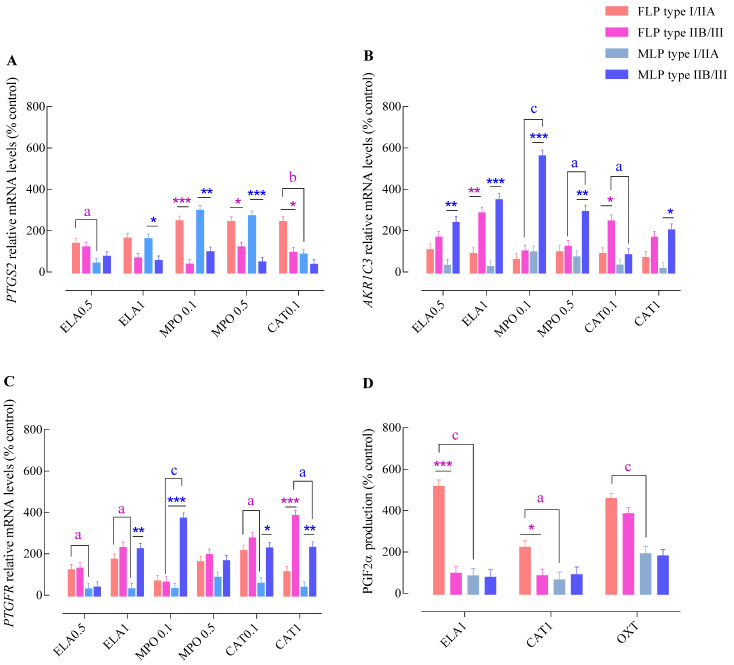
Effect of endometrial category (type I/IIA vs. type IIB/III) and estrous cycle phase (FLP—follicular phase FLP vs. MLP—mid-luteal phase) on *PTGS2* (**A**)*, AKR1C3* (**B**), or *PTGFR* (**C**) mRNA transcription and PGF_2α_ production (**D**) in endometrial explants, incubated with different concentrations of NETs components. ELA-elastase (0.5 or 1 mg/mL); MPO—myeloperoxidase (0.1 or 0.5 mg/mL); CAT—cathepsin G (0.1 or 1 mg/mL); and OXT—oxytocin. All values are expressed as percentage of change from control (non-treated tissues). Bars represent the least significant difference (LSD) means ± SEM. Asterisks indicate significant differences between endometrial category groups (I/IIA vs. IIB/III) within each estrous cycle phase (* *p* < 0.05; ** *p* < 0.01; and *** *p* < 0.001); a, b, and c—letters indicate significant differences between estrous cycle phases (FLP vs. MLP) within each endometrial category group (a—*p* < 0.05; b—*p* < 0.01; and c—*p* < 0.001).

In FLP, the highest *PTGS2* gene transcription was detected in I/IIA endometria stimulated with MPO (MPO 0.1 µg/mL—*p* < 0.001; MPO 0.5 µg/mL—*p* < 0.05) and the lowest dose of CAT (*p* < 0.05) (Figure 2A). In contrast, up-regulation of *AKR1C3* transcripts was only detected in type IIB/III endometrial explants incubated with ELA (1 µg/mL; *p* < 0.001) and CAT (CAT 0.1 µg/mL—*p* < 0.05; Figure 2B). Type IIB/III endometrial explants also exhibited increased *PTGFR* mRNA levels after stimulation with CAT (1 µg/mL—*p* < 0.001; Figure 2C), when compared to type I/IIA tissues. Up-regulation of PGF_2α_ production was noted in type I/IIA explants incubated with the highest concentrations of ELA (*p* < 0.001) and CAT, (*p* < 0.05) in comparison to type IIB/III group (Figure 2D).

In MLP, the highest *PTGS2* mRNA levels were found in type I/IIA endometrial explants stimulated with ELA (1 µg/mL—*p* < 0.05) or MPO (0.1 µg/mL—*p* < 0.01 and 0.5 µg/mL; *p* < 0.001; Figure 2A). In contrast, the highest transcript levels of *AKR1C3* were detected in type IIB/III endometrial explants challenged with all tested enzymes (CAT 1 µg/mL—*p* < 0.05; ELA 0.5 µg/mL or MPO 0.5 µg/mL—*p* < 0.01; and ELA 1 µg/mL or MPO 0.1 µg/mL—*p* < 0.001) (Figure 2B). Similarly, the type IIB/III endometrial explants presented a higher *PTGFR* transcription level after stimulation with ELA (1 µg/mL—*p* < 0.01), MPO (0.1 µg/mL—*p* < 0.001), and CAT (0.1 µg/mL—*p* < 0.05 and 1 µg/mL—*p* < 0.01), compared to type I/IIA tissues (Figure 2C). Nevertheless, no differences were detected in PGF_2α_ production between endometrial categories in MLP explants (*p* > 0.05; Figure 2D).

### 3.4. Influence of the Estrous Cycle Phase on the PGF_2α_—Pathway Activation by NETs Enzymes

The influence of different phases of mare estrous cycle (FLP vs. MLP) on the effect of NETs components in putative PGF_2α_ fibrogenic pathway was also analyzed. This evaluation was completed comparing the same treatments, in the same endometrium type (I/IIA or IIB/III).

In type I/IIA endometria, an up-regulation of *PTGS2* transcription was detected in FLP tissues compared to those in MLP after incubation with ELA (ELA 0.5 µg/mL—*p* < 0.05) and CAT (CAT 0.1 µg/mL—*p* < 0.001; Figure 2A). Nevertheless, no differences between estrous cycle phases were found in *AKR1C3* mRNA levels after stimulation with NETs proteases (*p* > 0.05; Figure 2B). Regarding *PTGFR*, increased mRNA levels were found in FLP explants incubated with ELA (ELA 0.5 μg/mL or ELA 1 μg/mL—*p* < 0.05) and CAT (CAT 0.1 µg/mL*—p* < 0.05; Figure 2C), when compared to MLP tissues. A marked increase in PGF_2α_ production was observed in FLP endometria incubated with ELA (1 µg/mL—*p* < 0.001), CAT (1 µg/mL—*p* < 0.05), and OXT (*p* < 0.001) compared with MLP explants (Figure 2D). 

In type IIB/III explants, no differences in *PTGS2* transcription levels (Figure 2A) were noticed between estrous cycle phases. In contrast, type IIB/III MLP explants showed increased levels of *AKR1C3* mRNA after stimulation with MPO (0.1 µg/mL—*p* < 0.001 or 0.5 µg/mL—*p* < 0.05) and CAT (0.1 µg/mL—*p* < 0.05) when compared with FLP endometria (Figure 2B). Type IIB/III MLP endometrial explants also showed a differential transcription of *PTGFR*. Challenge with MPO (0.1 µg/mL—*p* < 0.001) induced a higher transcription level of *PTGFR* by these endometria, and CAT (1 µg/mL—*p* < 0.05) resulting in a lower mRNA expression when compared with FLP tissues (Figure 2C).

In type IIB/III explants, no differences in PGF_2α_ production were seen between estrous cycle phases throughout the experiment (Figure 2D).

## 4. Discussion

The exact etiology and pathological mechanisms leading to endometrosis and subsequent progressive reproductive failure has not been fully elucidated. Nevertheless, this extremely complex pathology may be caused by persistent endometritis, which results in enhanced neutrophil recruitment to the uterus and NETs release [27]. In the human lung, when extracellular concentration of free ELA released by neutrophils exceeds the buffering capacity provided by endogenous inhibitors, ELA starts to drive uncontrolled inflammatory processes [50]. Likewise, the same may occur in the uterus of mares with NETs persistence. 

The study showed that PGF_2α_ pathway is activated in equine endometrial explants challenged in vitro by enzymes found in NETs. These results, in association with our previous studies on the same endometria [48], which were used in this follow-up work, further suggest an association between the PGF_2α_-pathway and collagen deposition in mare endometrium. Indeed, we were able to show that enzymes present in NETs could have a pathogenic role in collagen deposition on mare endometrium, as the in vitro incubation of endometrial explants with some of those enzymes induced high collagen type I (COL1) production [48]. In addition, previous in vitro studies in mare endometrium have already evidenced an association between COL1 and PGF_2α_ [51], namely after stimulation of endometrial explants with ELA [52].

In this study, the putative involvement of the PGF_2α_ pathway in mare endometrial fibrogenesis induced by NETs enzymes was observed in all studied types of endometria, except in MLP I/IIA endometria. In FLP tissues from all endometrial grades, the production of PGF_2α_ and/or transcription of PGF_2α_ receptor was increased when stimulated with ELA or CAT. This endometrial response to NETs enzymes is also associated with an increase in COL1 production by the same endometria, as we have previously shown [48], suggesting an association between the PGF_2α_ pathway and equine endometrial fibrosis development.

In contrast, in MLP healthier endometrium (type I/IIA), the PGF_2α_ pathway does not seem to be involved in the initiation of mare endometrial fibrogenesis induced by NETs. Other mechanisms besides the PGF_2α_ pathway may be involved, as high COL1 production, induced by ELA and CAT in this type of endometrial tissues [48], is associated with low *AKR1C3* or *PTGFR* gene expression in the present work. In this type of endometrium, the PGE_2_ pathway may facilitate fibrogenesis due to suppression of the antifibrotic action of PGE_2_ [53]. Nevertheless, in MLP type IIB/III endometrium, activation of the PGF_2α_ pathway appears to be essential to intensify the course of fibrogenesis. Moreover, all enzymes under study that induced COL1 production [48] showed high relative transcription of *PTGFR*, except with the lowest concentration of ELA, as now shown. This is in agreement with previous studies on lung and myocardium fibrosis, where PTGFR-PGF_2α_ has been shown to predispose for tissue fibrosis [13,20,22]. In addition, a selective human PGF_2α_ receptor antagonist whose efficacy has been proven in vivo in a preclinical animal model of pulmonary fibrosis has been developed [54].

In the present work, we have also investigated whether the effect of enzymes present in NETs on the PGF_2α_ pathway in mare endometrium could depend on endometrial fibrotic lesions. Endometrial category appears to influence the PG secretion, as enhanced *PTGS2* transcripts were detected in I/IIA groups, when compared with endometrial type IIB/III. These results are in agreement with previous studies in lung fibroblasts showing that lung fibrotic cells have a decreased ability to up-regulate *PTGS2* mRNA levels and an increased fibrogenesis capacity [55]. This agrees with the present work, where the stimulation of PGF_2α_ production by NETs proteases was only observed in FLP I/IIA endometrial tissues, as previously described [28]. 

Endometria graded as type IIB/III were able to respond to the enzymes present in NETs with higher *AKRC1* transcripts, regardless of the estrous cycle phase. Additionally, the type of endometrium seems to influence *PTGFR* mRNA in response to these proteins, as type IIB/III explants presented the highest *PTGFR* transcript levels. 

The putative endocrine influence on endometrial response to enzymes reported in NETs was addressed by assessing PGF_2α_ mediators in FLP and MLP tissues. As mentioned before, increased levels of PGF_2α_ synthesis (after ELA or CAT treatments) were only detected in FLP endometria. Such differences may be hormone-dependent and mediated by tissue specific catabolic or anabolic enzymes involved in PG synthesis. In vitro studies have shown that ovarian steroids not only trigger PG production, but also modulate mare endometrial cell response to interleukins [56]. In a previous work on steroid regulation of PG synthesis, after in vitro exposure of mare endometrium to estrogen, a dose-dependent stimulation of PGF_2α_ production was observed, while exposure of tissues to progesterone failed to alter PGF_2α_ secretion [57]. Likewise, endometrial explants collected from mares during the FLP exhibited a higher basal concentration of PGF_2α_ secretion, than explants collected during the luteal phase after 24 h or 72 h incubation [41]. Taken together, these effects suggest that, in the face of a stimulus, a higher production of both PG will occur in FLP endometria, rather than in MLP tissues, as physiological endometrial PG synthesis mechanisms are activated. 

In the mare, following the entrance of bacteria or semen into the uterus, the resulting recruitment of neutrophils will trigger the release of PGF_2α_ that induces uterine contractions [58], a crucial mechanism for clearance of the uterine fluid produced by an initial inflammatory response [59]. Mares predisposed to develop persistent endometritis (susceptible mares) have an increased number of neutrophils in the endometrium after breeding, when compared with resistant mares [60]. It has been suggested that the increased PGF_2α_ endometrial release in susceptible mares might be due to the sustained inflammatory uterine environment in these mares [61]. An altered cytokine response with up-regulation of *IL-1B*, *IL-6*, and *TNFα* transcripts, 24 h after breeding has been shown in susceptible mares in vivo [62]. In vitro studies have suggested that equine endometrial tissues are able to produce PG in response to different cytokines. As such, IL1α, IL1ß, and IL6 increased PGF_2α_ secretion from endometrial explants, regardless of Kenney and Doig´s endometrial category [40]. Indeed, PGF_2α_ has been proposed as a suitable marker of uterine inflammation during mating-induced endometritis in the horse [63]. In the mare, target tissues might be exposed to higher concentrations of PGF_2α_ for longer periods, due to its low plasma clearance [64]. In addition, an endometrial PGF_2α_ auto-amplification system appears to exist, in which PGF_2α_ can stimulate its own production [65]. Therefore, these physiological features may contribute to sustained high levels of PGF_2α_ in the uterus. Thus, we propose that the presence of an insult in the mare endometrium, such as the deleterious effect of enzymes present in NETs, will trigger PGF_2α_ leading to in loco fibrogenesis.

Previous studies have suggested that sex steroid hormones that regulate the reproductive cycles modulate the expression of PG receptors such as PGE_2_ receptors and PTGFR [66]. Nevertheless, in the mare endometrium, *PTGFR* transcription may not be influenced by the endocrine cyclic changes, as *PTGFR* mRNA levels were similar at day 7 (MLP), 14 (late-luteal phase), and 21 (estrus) of the estrous cycle [67]. In accordance with the physiological condition, our results also suggest that the mechanisms involved in *PTGFR* gene transcription are not estrous cycle phase-dependent, as *PTGFR* transcription was up-regulated in both FLP and MLP endometrial tissues, under the action of enzymes found in NETs.

In the mare, it has been long recognized the luteolytic physiological role of PGF_2α_ on a well-established corpus luteum. However, to the best of our knowledge no in vivo evidence of PGF_2α_ fibrogenic pathological effect has been reported in mare endometrium [68]. While multiple low doses of PGF_2α_ in early luteal phase (ELP) have antiluteogenic effects, a single dose of PGF_2α_ can interrupt luteal function in the MLP [69]. In the present study, increased in vitro endometrial PGF_2α_ output in response to enzymes present in NETs was only observed in FLP. Furthermore, as increased *PTGFR* mRNA was detected in the endometrium, but it was not evaluated in the corpus luteum, we cannot speculate whether these antiluteogenic or luteolytic effects, ascribed to PGF_2α_, can be induced by NETs enzymes in either ELP or MLP.

Even though our results have suggested that the PGF_2α_ pathway may be involved in COL1 formation in mare endometrium, induced by NETs enzymes, other mechanisms, such as the PGE_2_ pathway [53] and pro-fibrotic cytokines, may be involved. Indeed, it has been questioned if PGF_2α_ stimulates fibrogenesis through a TGF-β1 dependent [23] or independent mechanism [13,20,21,24]. However, the present in vitro data on the effect of NETs enzymes on mare endometrial fibrogenesis might not be directly extrapolated to in vivo studies, as mentioned by others [70]. Despite these considerations, our in vitro data should be considered as a useful initial approach to further pursue in vivo studies on putative fibrotic pathways.

## 5. Conclusions

In conclusion, our results indicate that FLP endometrium might be less protected from pro-fibrotic mediators. In fact, it is at this stage that we have observed the highest PGF_2α_ production and/or *PTGFR* mRNA levels under the action of enzymes found in NETs, both associated with fibrogenesis in other tissues. The transcription of the *PTGFR* gene does not appear to be hormone-dependent, although their levels seem to be dependent on endometrial category in the MLP, shaping fibrosis outcome. Although other pro-fibrotic cytokines may be involved, this study suggests that enzymes existing in NETs may instigate changes on PGF_2α_ mediators, which may become an additional mechanism of fibrogenesis in mare endometrium. Additionally, the endocrine microenvironment of healthy and pathological endometrium might modulate the PGF_2α_-pathway, as well as fibrosis development on endometrium challenged by NETs enzymes. Thus, further studies are needed to elucidate if putative fibrogenic PGF_2α_ may act in vivo in the mare endometrium, through a cytokine dependent or independent mechanism.

## Figures and Tables

**Table 1:** Primer sequences used in real time PCR analysis.

Gene (Acession Number)	Sequence 5′-3	Amplicon (Base Pairs)
*AKR1C3* (XM_001500921.1)	Forward: TGGGTCACTTTCCTTCAACCA	200
	Reverse: CTTCTCCATTGCCTCCCATGT	
*PTGFR* (NM_001081806.3)	Forward: GTGCAATGCCATCACAGGAA	225
	Reverse: GCCATTCGGAGAGCAAACAG	
*PTGS2* (NM_001081775.2)	Forward: TGCTGTTCCAACCCGTGTC	204
	Reverse: GACAATGTTCCAGACTCCCTTGA	
*RPL32* (XM_001492042.6)	Forward: AGCCATCTACTCGGCGTCA	144
	Reverse: GTCAATGCCTCTGGGTTTCC	

*AKR1C3—*aldo-keto reductase family 1, member C3; *PTGFR—*prostaglandin F_2α_ receptor; *PTGS2*—prostaglandin-endoperoxide synthase 2; and *RPL32*—ribosomal protein L32.

**Table 2:** Two- and three-way interactions and significance levels (*p* values) between estrous cycle phases, endometrial category groups, and treatments for relative mRNA levels of PGF_2α_ pathway target genes and PGF_2α_ production by equine endometrial explants.

Interactions	*PTGS2*	*AKR1C3*	*PTGFR*	PGF_2α_
estrous cycle X endometrial categories	0.6387	<0.0001	0.0008	<0.0001
estrous cycle X treatments	0.0004	<0.0001	<0.0001	<0.0001
endometrial categories X treatments	<0.0001	<0.0001	<0.0001	0.003
estrous cycle X endometrial categories X treatments	0.2969	<0.0001	0.00005	0.005

*PTGS2*—prostaglandin-endoperoxide synthase 2; *AKR1C3—*aldo-keto reductase family 1, member C3; *PTGFR—*prostaglandin F_2α_ receptor; and PGF_2α_—prostaglandin F_2α._

## Data Availability

Data will be available upon request to the corresponding author.
